# Case Report: Novel combinatorial factors in the WNT pathway in a pediatric case of valvular aortic stenosis from Lebanon: a brief report

**DOI:** 10.3389/fcvm.2025.1614666

**Published:** 2025-10-07

**Authors:** Wiam Ataya, Fathima Mohammed, Mariam Arabi, Fadi Bitar, Georges Nemer

**Affiliations:** ^1^ Faculty of Medicine, Beirut Arab University, Beirut, Lebanon; ^2^ College of Health and Life Sciences, Hamad Bin Khalifa University, Doha, Qatar; ^3^ Department of Pediatrics and Adolescent Medicine, American University of Beirut Medical Center, Beirut, Lebanon; ^4^ Department of Biochemistry and Molecular Genetics, American University of Beirut, Beirut, Lebanon

**Keywords:** aortic stenosis, consanguinity, whole-exome sequencing, wnt signaling pathway, polygenic inheritance, congenital heart disease, case report

## Abstract

**Introduction:**

Aortic stenosis (AS) is a common valvular disease with a complex and incompletely defined genetic architecture. The contribution of inherited factors may be particularly prominent in consanguineous populations, where familial clustering suggests a strong hereditary component. We investigated the genetic basis of AS in a consanguineous Lebanese family.

**Methods:**

We performed clinical phenotyping and trio whole-exome sequencing (WES) on a 12-year-old female proband with severe valvular AS and her phenotypically normal consanguineous parents. Variants were assessed with standard filtering for rarity, predicted functional impact, and biological plausibility, with particular attention to genes implicated in cardiovascular development and signaling pathways.

**Results:**

The proband presented with severe aortic stenosis, bicuspid aortic valve, dilated aortic root and ascending aorta, and mild -moderate tricuspid regurgitation, requiring multiple interventions (balloon valvuloplasty, Ross procedure, and right ventricle-pulmonary artery conduit replacement). WES identified three heterozygous variants in genes belonging to the Wnt signaling pathway *APCDD1*, *DVL1*, and *AXIN2* in the proband, which were inherited from the normal parents.

**Discussion:**

The co-occurrence of heterozygous variants in Wnt pathway genes in a child with severe AS highlights a potential polygenic or pathway-level contribution to disease susceptibility, even within a consanguineous context. These findings support a role for Wnt signaling in aortic valve development and pathology, motivating further functional studies and broader cohort analyses to clarify pathogenicity, segregation, and clinical relevance.

## 1 Introduction

Aortic stenosis (AS) is a valvular disease characterized by restricted blood flow through the aortic valve and narrowing of the left ventricular outflow tract often leading to left ventricular systolic dysfunction (1). Early onset AS is associated with gene mutations affecting valvulogenesis (2), which can lead to more severe phenotypes and an earlier onset of AS. In Lebanon, consanguinity rates exceed 35% in some areas (3), increasing the likelihood of autosomal recessive mutations (4) that can cause AS. The genetics of familial AS in consanguineous populations Remains poorly characterized. This study aims to identify novel pathologic variants underlying this disease.

## 2 Case presentation

### 2.1 Patient information

**Figure 1:**
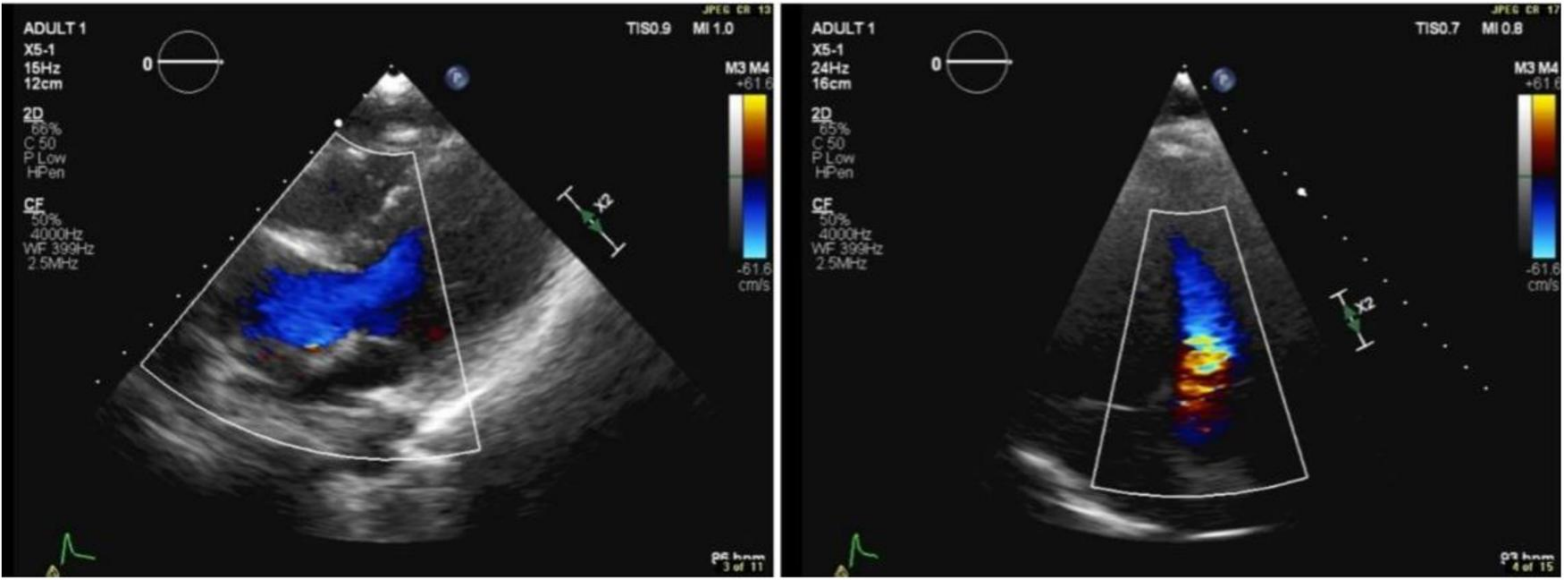
Transthoracic echocardiographic color Doppler showing flow turbulence through the RV-PA conduit. Peak gradient: 25 mmHg; mean gradient: 13 mmHg.

We report the case of a 12-year-old female diagnosed with severe aortic stenosis and a bicuspid aortic valve, with the only child of phenotypically normal consanguineous parents. The patient had progressive aortic valve dysfunction and calcification from an early age, requiring multiple interventions (Figure 1). In June 2009, she underwent balloon valvuloplasty, which was initially effective with only mild residual stenosis. However, further valvular dysfunction required a Ross procedure in February 2015, during which the aortic valve was replaced with her native pulmonary valve. A 16 mm Contegra conduit was placed to replace the right ventricular outflow tract, and a 20 mm Dacron tube graft was used to replace the ascending aorta. In March 2016, the patient required an 18 mm RV-PA conduit replacement to restore right ventricular outflow tract continuity due to calcification of the previous one.

### 2.2 Diagnostic workup

In April 2018, transthoracic echocardiography was performed as part of the patient's follow-up. The imaging revealed a dilated aortic root, with the sinus of Valsalva measuring 3.1 cm. Some cardiac valve anomalies were present, including mild-to-moderate tricuspid regurgitation (TR), and trace mitral regurgitation (MR). There was no evidence of shunting at the atrial, ventricular, or arterial levels. The other cardiac chambers are grossly normal in size, thickness, and systolic function and hemodynamic assessment showed increased flow velocity at the level of the conduit to the pulmonary artery branches, with a peak systolic gradient of 25 mmHg and a mean gradient of 13 mmHg. Right ventricular (RV) function remained within normal limits, and there were no signs of pericardial effusion or aortic coarctation.

### 2.3 Genetic findings

Whole-exome sequencing (WES) was performed on genomic DNA extracted from peripheral blood samples of the patient and her parents (the only three individuals analyzed in this study). A stringent variant filtering strategy was implemented, adhering to the American College of Medical Genetics and Genomics (ACMG) guidelines for variant interpretation and classification, and informed by a comprehensive literature review to identify optimal filtering criteria for whole-exome sequencing (WES) analyses in rare congenital heart diseases. Initially, homozygous variants predicted to be pathogenic or likely pathogenic (based on ACMG categories PVS1, PS1-PS4, PM1-PM6, PP1-PP5, and absence of benign criteria BA1-BS4) and with a minor allele frequency (MAF) below 1% were screened to detect potential autosomal recessive inheritance. However, no homozygous deleterious variants meeting these stringent criteria were found in this family. Relevant *de novo* variants were also excluded from subsequent analyses. Analyses then focused on identifying compound heterozygous mutations within individual candidate genes. Despite a thorough assessment, none of the detected compound heterozygous variants within single genes met the ACMG criteria or were supported by literature evidence as disease-causing for congenital heart defects. Therefore, further analyses examined compound heterozygous variants affecting multiple genes within the same biological pathway. This rigorous filtering approach resulted in the identification of three variants (Table 1) across genes implicated in cardiac development: a maternally inherited variant in *APCDD1* (adenomatosis polyposis coli down-regulated), and paternally inherited variants in *AXIN2 (*axis inhibition protein 2) and *DVL1* (Disheveled segment polarity protein 1).

**Table 1:** Filtered-Genetic variants.

Gene	Variant	Chr	Coordinate	Type	Genotype	Exonic	Inherited From	Read Depth	dbSNP ID	Sift	PolyPhen	CADD Score
APCDD1	C>C/T	18	1,0487,749	snv	het	Yes	Mother	92	rs114154601	Deleterious (0.04)	Probably damaging (0.993)	24.2
DVL1	G>G/A	1	1,275,810	snv	het	Yes	Father	76	rs747373290	Deleterious (0)	Probably damaging (0.942)	26.8
AXIN2	G>G/A	17	63,532,604	snv	het	Yes	Father	55	rs142670753	Deleterious (0.02)	Possibly damaging (0.885)	28.1

These three genes have been found to be implicated in the valvular developmental under the WNT signaling pathway, implying that a single or combination of these genes could contribute to the patient's aortic stenosis phenotype.

## 3 Discussion

This study describes the genetic basis of valvular aortic stenosis in one consanguineous Lebanese family and has identified novel variants in *APCDD1*, *AXIN2*, and *DVL1*, which collectively might explain the phenotype.

Previous studies have linked aortic stenosis to mutations in multiple different genes, most noticeably NOTCH1which was highly correlated with AS given its important role in valvulogenesis and cardiac development (5). Other studies have also identified Lp(a), which is involved in lipid transport, as well as IL6, which is implicated in multiple different inflammatory pathways, as contributors to AS pathogenesis (6, 7), while several others have associated AS to the WNT signaling pathway (8) thus, in addition to valvular calcification and inflammation, alternative pathways involving extracellular matrix remodeling and WNT signaling may contribute to the development AS which emphasizes the genetic heterogeneity of the disease and the need for further research in this field.

**Figure 2:**
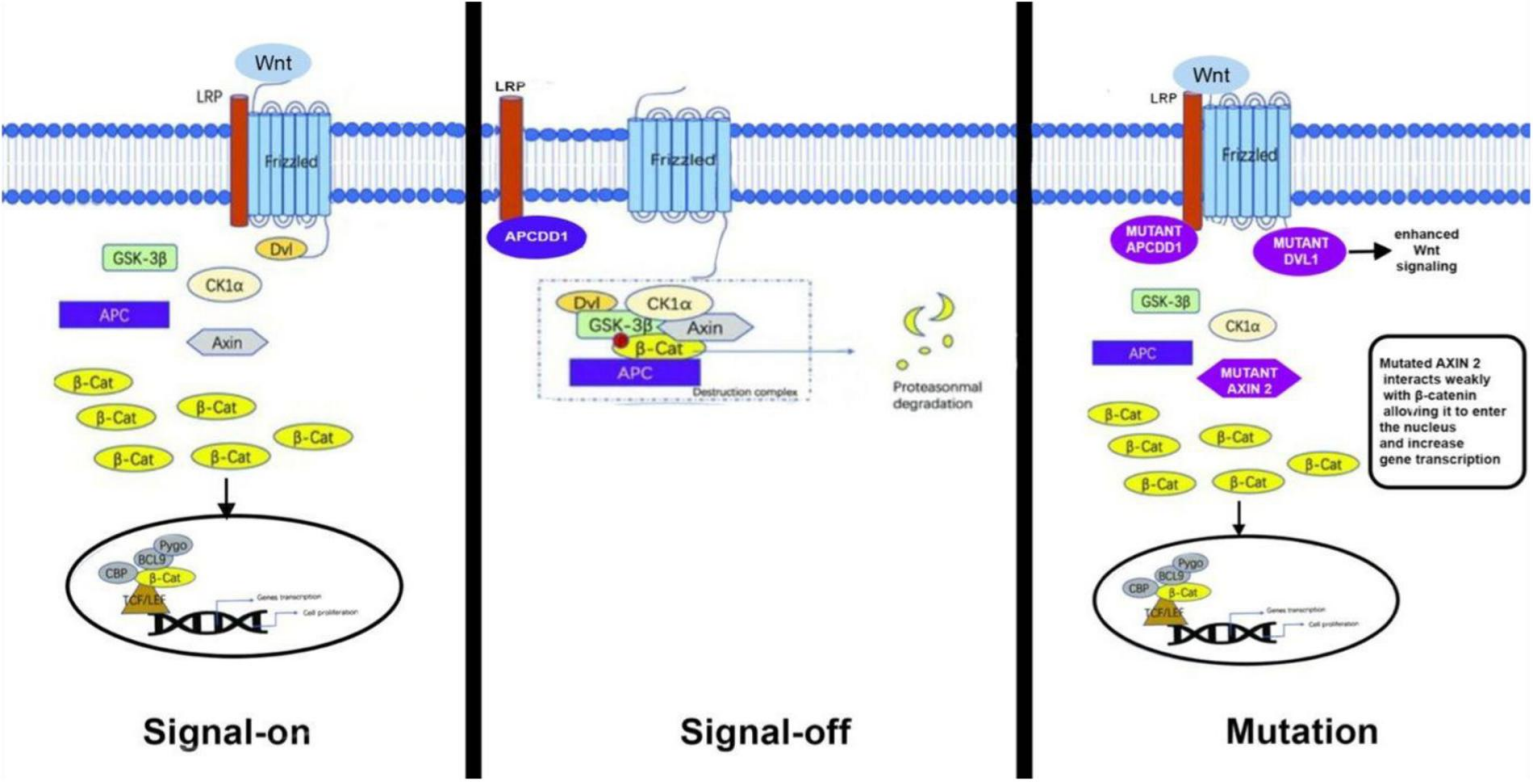
Schematic diagram of the canonical WNT signaling pathway.

In our case, compound heterozygous mutations were detected in *APCDD1*, *AXIN2*, and *DVL1*. Although no variant alone was classified as pathogenic, the presence of heterozygous mutations in APCDD1, AXIN2, and DVL1, all of which are implicated in WNT signaling, suggests a polygenic pathogenesis of AS with a combinatorial inheritance from the parents. In the context of congenital aortic stenosis, the identified missense variants in *DVL1, AXIN2,* and *APCDD1* collectively suggest an intriguing mechanism implicating enhanced WNT signaling activity. Specifically, the missense variant in *DVL1* may enhance its affinity toward the Frizzled (Fz) receptor, thereby potentiating WNT pathway activation through stabilized receptor interaction and downstream signaling. Concurrently, the variant identified in *AXIN2* potentially reduces its affinity for β-catenin, impairing the effective assembly of the β-catenin degradation complex. This decreased affinity can result in elevated cytoplasmic β-catenin levels, facilitating nuclear translocation and enhanced transcription of WNT-responsive genes involved in valvular interstitial cell proliferation and matrix remodeling. Similarly, the missense variant in *APCDD1* may result in reduced affinity toward LRP5/6 co-receptors, diminishing its inhibitory capacity on WNT signaling (Figure 2). Collectively, these subtle molecular alterations—enhanced DVL1-Fz interaction, diminished AXIN2–β-catenin interaction, and attenuated APCDD1–LRP5/6 binding—could synergistically amplify canonical WNT signaling. Such a mechanism aligns well with documented evidence that dysregulated WNT signaling contributes to pathological valve thickening, fibrosis, and calcification—hallmarks of aortic stenosis (9).

These findings emphasize the delicate balance within the WNT signaling pathway critical for normal aortic valve formation and underscore how slight functional changes in pathway components can collectively predispose individuals to congenital valve malformations, including aortic stenosis.

These results support the developing pattern of polygenic inheritance patterns in congenital heart diseases (CHD)where different variants within the same functional pathway act synergistically and increase disease risk (10). This becomes especially relevant in consanguineous populations where deleterious mutations become more apparent and may be at an increased risk of developing CHD.

Uncovering the genetic variants of AS has major potential in the field of precision medicine, especially in high-risk groups, including consanguineous families despite the very limited outcomes for the autosomal recessive pattern postulate. Genetic screening could provide early diagnosis of AS and enable the development of specific personalized therapies.

## 4 Strengths and limitations

Our study provides insights into the genetic basis of AS through whole exome sequencing allowing for fast and accurate variant identification. Furthermore, this study adds to the limited research and literature on the genetic variants of valvular aortic stenosis. However, the small sample size (a single affected patient analyzed with her parents) limits the generalizability of the results. No other consanguineous families with AS or unrelated controls were included, so the proposed contribution of WNT pathway variants to this patient's phenotype remains speculative. Future studies involving larger cohorts and functional analyses are planned to validate these preliminary findings. Additionally, undetected genetic or epigenetic changes may influence disease severity, as our study did not investigate non-coding regions of the DNA.

## 5 Conclusion

This study broadens the genetic outlook of AS by confirming the WNT pathway through combinatorial interaction as a major player in congenital heart diseases. These findings point out the complexity of AS genetics, the interplay of multiple different genetic pathways in its pathology, and the need for continued research into its genetic basis. Future studies involving additional patients are warranted to validate these WNT-related genetic findings and confirm their contribution to AS pathogenesis.

## Data Availability

The datasets presented in this study can be found in online repositories. The names of the repository/repositories and accession number(s) can be found below: https://www.ncbi.nlm.nih.gov/, PRJNA1237610.
